# Impact of operative timing for proximal humerus fracture dislocations on development of neurologic injury

**DOI:** 10.1007/s00590-026-04956-y

**Published:** 2026-09-12

**Authors:** Alex Demers, Maria Bozoghlian, Natalie Glass, Brendan Patterson, James Nepola, Matthew Hogue, Michael Willey, Matthew Karam, J. Lawrence Marsh, Joseph Galvin

**Affiliations:** Department of Orthopaedics and Rehabilitaiton, University of Iowa, Iowa City, USA

**Keywords:** Proximal humerus fracture, Dislocation, Neurologic injury

## Abstract

**Purpose:**

Operative management of proximal humerus fractures with glenohumeral dislocation is often delayed until a shoulder specialist is available. This study aims to assess whether time from presentation to operative intervention impacts incidence of neurologic injury in the preoperative waiting period.

**Methods:**

Retrospective review was performed for patients undergoing operative management of a proximal humerus fracture dislocation at a level 1 trauma center. Patient charts were reviewed for patient factors, injury characteristics, neurologic status, and postoperative complications. Statistical analysis comparing patients undergoing operative intervention ≤ 5 and > 5 days from injury was performed (α ≤ 0.05).

**Results:**

Of 52 proximal humerus fracture dislocations, 14% had a neurologic deficit at presentation, and 1.9% had a change in neurologic status prior to operative intervention. 32 injuries (61.5%) underwent operative intervention ≤ 5 days from presentation and 20 (38.5%) > 5 days. There was no difference in neurologic deficit at time of presentation (18.8% vs. 10.0%, *p *=.077), incidence of preoperative neurologic exam change (0% vs. 5.0%, *p *=.38), or residual neurologic deficit at final follow-up (9.4% vs. 5.0%, *p* = 1.0) for the ≤ 5 or > 5 day cohorts, respectively.

**Conclusion:**

Through this exploratory retrospective study, no differences were identified for rates of neurologic injury development in the preoperative waiting period between patients undergoing operative intervention ≤ 5 days or > 5 days from presentation for proximal humerus fractures with glenohumeral dislocation, however, this is limited by only 1 patient experiencing a preoperative neurologic change in the > 5 days group. Given the rarity of neurologic injury in the preoperative waiting period and low power of this study, future larger prospective studies are needed to better define the impact of waiting for operative intervention for these injuries.

**Level of Evidence:**

Level IV Case Series.

## Introduction

Proximal humerus fractures account for 5.7% of all adult fractures and have an increased incidence since 2000 [1, 2]. Proximal humerus fracture dislocations represent a rare subset of these injuries often associated with high-energy mechanisms and risk of neurovascular injury [3]. These injuries present surgical challenges due to high rates of reoperation and avascular necrosis (AVN) with rates of 15-35.6% and 14–21% respectively [3–9]. Management decisions regarding timing of fixation and choice of surgical management are made difficult due to potential risks of brachial plexopathy from the displacement of the humeral head [3]. Some studies have estimated a neurovascular injury rate of 0.9-5%, while others have reported neurologic injury rates of 6.2 to 67% following proximal humerus fractures, however, data remains limited for proximal humerus fracture dislocations [10–13].

A lack of consensus exists for the ideal timing for operative management of proximal humerus fracture dislocations, as there remains a paucity of studies examining impact of time from presentation to operative intervention on complication rates [14, 15]. While some 2-part fracture dislocations can be reduced in the emergency department, many complex injuries such as those with a completely dislocated humeral head fragment require reduction in the operating room along with definitive surgical management [3, 16, 17]. This presents a challenge for institutions who do not always have a shoulder or trauma specialist available to manage these injuries urgently. No studies have specifically examined the impact of timing of surgical intervention on neurologic injuries following proximal humerus dislocations [3–9, 18–20].

This retrospective study seeks to examine whether time from presentation to operative intervention impacts incidence of neurologic injury in the preoperative waiting period for proximal humerus fractures with associated glenohumeral dislocation. The secondary aim of this study is to compare rates of residual neurologic deficit, wound complications, reoperation, and mortality for proximal humerus fractures with glenohumeral dislocation for patients undergoing operative intervention ≤ 5 and > 5 days from presentation.

## Methods

A retrospective review was performed to identify patients with a proximal humerus fracture dislocation undergoing operative intervention at a level I academic trauma center from January 2018 to March 2025. Inclusion criteria were patients of any age with radiographs confirming presence of a proximal humerus fracture with glenohumeral dislocation. Patients with glenohumeral joint subluxation, simple shoulder dislocations, isolated glenoid fractures, isolated Hill-Sachs lesions, and those who underwent nonoperative management were excluded from analysis. Fractures that had successful closed reduction of their glenohumeral dislocation in the emergency department were excluded from final analysis, as a reduced glenohumeral joint would theoretically decrease the risk of brachial plexopathy and risk of avascular necrosis compared to injuries that remained dislocated.

Study methodology and execution were approved by our institutional review board (IRB) (IRB ID# 202501516) and need for patient consent was waived due to the retrospective nature of this study. Electronic medical records (EMR) were reviewed for patient factors including demographics, comorbidities, and body mass index (BMI). Injury factors including mechanism, polytrauma status, neurovascular status, concomitant injuries, American Society of Anesthesiologists Physical Status Score (ASA), and presenting lactate were recorded [21]. Injuries were categorized with low energy mechanisms including ground level fall (GLF), GLF/seizure, and fall from standing height or less. High energy injuries included motor vehicle crash (MVC), assault, and electrocution. Closed reduction attempts in the ED were noted. Call staff fellowship training was recorded from the orthopaedic consult note. Providers with no shoulder or trauma fellowship training were grouped together and labeled as ‘other specialty.’ EMR notes were reviewed to identify changes in neurologic exam documented by residents and confirmed by fellowship trained surgeons prior to operative interventions. For patients intubated or incapacitated on presentation, neurologic status was listed as unknown. Shoulder radiographs and computed tomography (CT) scans were reviewed to determine the modified Neer’s classification and Orthopaedic Trauma Association/Arbeitsgemeinschaft für Osteosynthesefragen (OTA/AO) fracture and dislocation classification for each injury [17, 22, 23]. Time to surgical intervention was determined from the time of presentation to procedure in days. Patients with residual neurologic injury were noted along with electromyography acquisition and neurologic intervention.

The individual shoulder served as the unit of analysis because patients with bilateral injuries could have different physical examination findings, operative management, and clinical status at the time of surgery for each shoulder. Shoulders were stratified into one of two groups by the time from presentation to surgery as ≤ 5 days or > 5 days. 5 days was chosen as time cutoff as this was deemed to be the worst case wait time at our institution for specialized care such as shoulder arthroplasty, when fellowship expertise and operating room access may not be readily accessible. Descriptive statistics were performed for all patient, injury, operative, and postoperative factors for the cohort as a whole and by group. Chi-square or Fisher’s Exact tests were used to compare qualitative variables between groups, as appropriate. Distributions of continuous variables were evaluated for normality using Shapiro-Wilk tests as well as through visual inspection of histograms. Independent t-tests or Wilcoxon Rank Sum tests were used to compare continuous variables with or without normal distributions, respectively, between groups. Statistical analyses were performed in SPSS 29 (IBM Armonk, NY). P value ≤ 0.05 was defined as statistical significance.

## Results

**Fig. 1:**
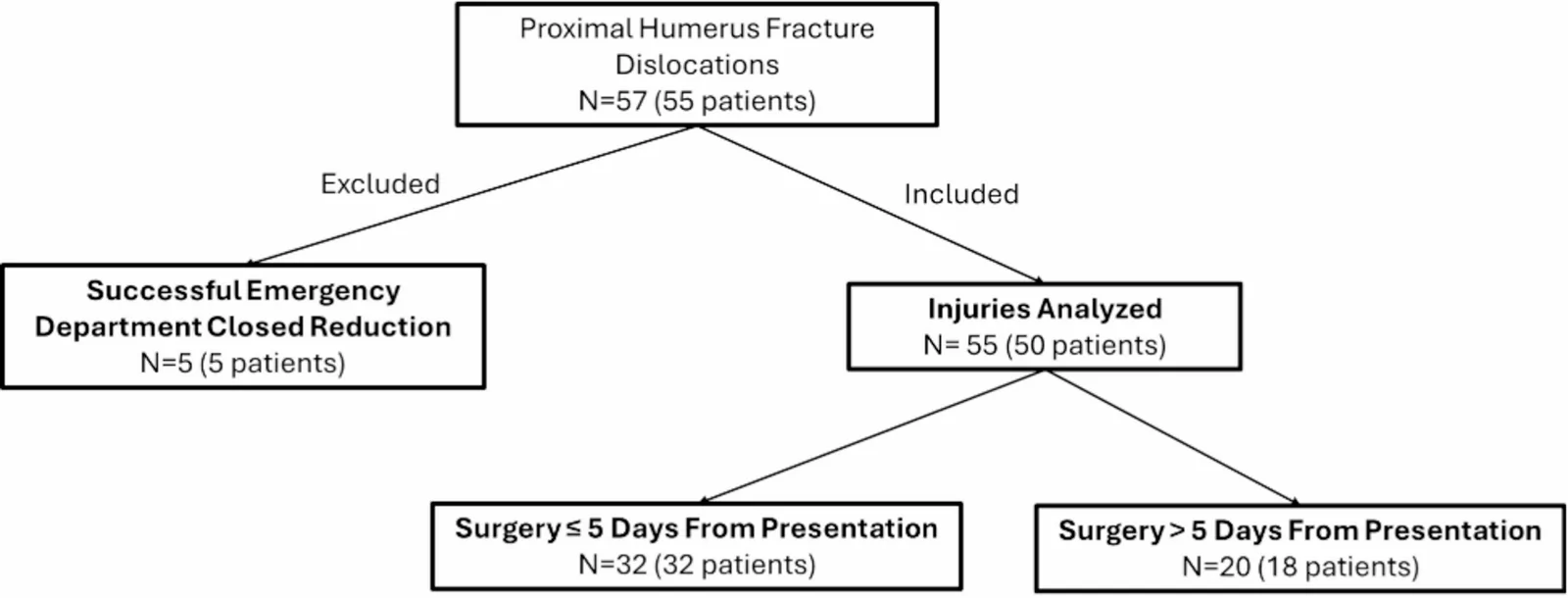
Flow diagram demonstrating breakdown of patient cohorts

We identified 55 patients sustaining 57 proximal humerus fractures with associated glenohumeral dislocation meeting inclusion criteria. Average age of the cohort was 59.0 ± 19.0 years with 60% being female and an average BMI of 29.3+/-6.7 kg/m^2^. 77.2% of the injuries were anterior fracture dislocations with the majority classified as Neer type 13 (4-part fracture dislocation) at 56.1% and OTA/AO 11C3.2 injuries at 57.9%. Ground level fall was the most common injury mechanism at 52.6%. 14% presented with a neurologic deficit and 5.3% had an unknown exam on presentation. For patients presenting with neurologic injury, three presented with a combined sensory and motor axillary nerve palsy, two with a sensory axillary nerve palsy, two with a sensory axillary, median, ulnar, radial nerve deficit, and one with a sensory ulnar nerve palsy. 50.9% of injuries presented when a shoulder or trauma surgeon was not on call. Twelve patients had an attempted reduction in the emergency department with a successful reduction rate of 41.7%. 5 patients with successful reduction were excluded in the analysis of operative timing (Fig. 1). Two patients (3.5%) from the cohort experienced a change in neurologic status prior to operative intervention. One patient with a neurologic change experienced hypoesthesia in the axillary nerve distribution following successful closed reduction in the ED prior to undergoing ORIF 5 days after injury and was excluded from analysis.

Reverse total shoulder arthroplasty (RTSA) was the most common operative intervention at 42.1% followed by ORIF at 36.8%. Two patients (3.5%) presenting when no shoulder or trauma staff were available underwent closed reduction in the operating room. For subsequent surgical procedures, two patients who underwent index open reduction went on to undergo staged ORIF with rotator cuff repair and ORIF, respectively. One patient underwent lesser tuberosity ORIF with biceps tenodesis and rotator cuff repair following index closed reduction. These were considered planned reoperations and were excluded from outcome analysis. Overall reoperation rate of the cohort was 15.8%. Of those requiring unplanned reoperation, indications included greater tuberosity nonunion requiring ORIF, I&D with antibiotic spacer for fracture related infection, core decompression with hardware removal and manipulation under anesthesia for AVN, reverse total shoulder arthroplasty following closed reduction, hardware removal and conversion to hemiarthroplasty for AVN, and hardware removal for screw pullout. 7.0% of the cohort demonstrated a residual neurologic deficit at final follow-up with 3.5% electromyography acquisition. Mortality was 10.5% with an overall average follow-up of 10.1 +/-10.2 months (range: 0.6 to 45.7 months).

For patients undergoing operative intervention at ≤ 5 days and > 5 days of injury, no statistically significant differences were detected for demographics, comorbidities, or BMI (Table 1). No differences were identified between groups for mechanism of injury, open injury, polytrauma, ICU admission, or pressor requirement at presentation (Table 2). No differences were identified for Neer’s fracture classification, OTA/AO classification, or rates of attempted closed reduction in the ED (Table 2). For patients undergoing intervention ≤ 5 days, on call surgeons without shoulder or trauma training were on call 46.9% of the time in comparison to 50.0% for the > 5 day group (Table 2). No difference was identified for presenting neurologic status (*p*=.08), however the ≤ 5 days to operative intervention group had an insignificantly higher proportion of patients with a neurologic deficit at presentation at 18.8% compared to > 5 days at 10.0% (Table 2). The > 5 days group had an insignificantly higher proportion of patients with unknown neurologic exam at 15.0% (Table 2). One patient in the > 5 days group demonstrated a change in neurologic status in the preoperative period (Table 2). This patient with a change in neurologic status in the > 5-day group had onset of a combined sensory and motor axillary and radial nerve palsy. This patient was noted to have residual neurologic deficits at follow-up with expectant management at 1.6 months. No differences in type of operative intervention were identified (Table 2). There were no differences in reoperation, wound complications, residual neurologic deficit, subsequent EMG, or mortality (Table 3). One patient in each cohort underwent reoperation for AVN, with the patient in the ≤ 5 days group being revised to a hemiarthroplasty and the > 5 days patient undergoing core decompression. For residual neurologic deficits at final follow-up, two continued to have axillary nerve sensory and motor deficits, one with isolated axillary nerve weakness and one with axillary nerve weakness and neuropathic pain. The two patients who had EMG obtained demonstrated a chronic axillary nerve injury and upper trunk brachial plexopathy with muscular denervation with evidence of reinnervation, respectively.

**Table 1:** Pre-operative characteristics in patients with surgery ≤ 5 vs. > 5 days from time of presentation

Measure	Surgery > 5 days from presentation		*p*-value
	No (*n* = 32 Patients)	Yes (*n* = 18 Patients)	
Age (years)	61.5(45.0-74.5) Min-max = 21–96	63.0 (45.0–73.0) Min-max = 31–82	0.9034
Race:			
White	31 (96.9%)	17 (94.4%)	1.0000
African American	1 (3.1%)	1 (5.6%)	
Sex:			
Female	18 (56.3)	11 (61.1)	0.7382
Male	14 (43.8)	7 (38.9)	
Body Mass Index (kg/m^2^)	29.6 (24.1–32.5)	28.0 (23.2–31.6)	0.5922
Comorbidities:			
Coronary Artery Disease	2 (6.3%)	1 (5.6%)	1.0000
Congestive Heart Failure	2 (6.3%)	1 (5.6%)	1.0000
Chronic Obstructive Pulmonary Disease	2 (6.3%)	3 (16.7%)	0.3363
Chronic Kidney Disease	2 (6.3%)	0	0.5298
Osteoporosis	4 (12.5%)	2 (11.1%)	1.0000
Hypothyroidism	2 (6.3%)	3 (16.7%)	0.3363
Diabetes	2 (6.3%)	5 (27.8%)	0.0825
Asthma	0	2 (11.1%)	0.1249
Alcohol Abuse	8 (25.0%)	5 (27.8%)	1.0000
Active Tobacco Use	6 (18.8%)	7 (38.9%)	0.1797
Other Drug Use	1 (3.1%)	0	1.0000

**Table 2:** Comparison of injuries and perioperative factors for surgery ≤ 5 vs. > 5 days from time of presentation

Measure	Surgery > 5 days from presentation		*p*-value	
	No (*n* = 32 Injuries)	Yes (*n* = 20 Injuries)		
Laterality of Injury (n, % right)	15 (46.9%)	9 (45.0%)	1.0000	
Mechanism				
≤ standing height	22 (68.75)	14 (70.00)	0.7581	
> standing height	0 (0)	1 (5.00)		
Motor Vehicle Crash/Motor Cycle Crash	9 (28.13)	5 (25.00)		
Other	1 (3.13)	0		
Open Injury	0	1 (5.0%)	0.3846	
Prior Instability	0	0	-	
Polytrauma	6 (18.9%)	5 (25.0%)	0.7300	
ICU Admission	4 (12.5%)	6 (30.0%)	0.1562	
Average Presenting Lactate (mmol/L)	2.7 ± 1.5 (*n* = 15)	3.1 ± 1.3 (*n* = 10)		
Pressor Requirement	0	2 (10.0%)	0.1433	
Neurologic Status				
Intact	26 (81.25)	15 (75.00)	0.0769	
Neurologic Deficit	6 (18.75)	2 (10.00)		
Unknown	0	3 (15.00)		
Neer’s Classification				
6	1 (3.13)	1 (5)	0.6078	
7	1 (3.13)	1 (5)		
10	3 (9.38)	1 (5)		
11	0	2 (10)		
13	20 (62.50)	10 (50)		
14	7 (21.88)	5 (25)		
OTA/AO Classification				
11A3.3	0	1 (5)	0.2089	
11B3	2 (6.25)	2 (10)		
11C3.1	9 (28.13)	2 (10)		
11C3.2	15 (46.88)	14 (70.00)		
11C3.3 NV	5 (15.63) 1 (3.13)	1 (5.00) 0		
Call Staff Fellowship Training				
Trauma	10 (31.25)	5 (25.00)	0.1872	
Shoulder	7 (21.88)	5 (25.00)		
Other	15 (46.87)	10 (50.00)		
Attempted ED Closed Reduction	3 (9.4%)	2 (10.0%)	1.0000	
Change in Neurologic Status Prior to Surgery		0 (0%)	1 (5.00%)	0.3846
American Society of Anesthesiologists Score:				
1		1 (3.13%)	0 (0.00%)	0.5248
2		13 (40.63%)	10 (50.0%)	
3		16 (50.00%)	7 (35.0%)	
4		2 (6.25%)	3 (15.0%)	
Closed Reduction		2 (6.3%)	0	0.5173
Open Reduction		14 (43.8%)	6 (30.0%)	0.3214
ORIF		12 (37.5%)	6 (30.0%)	0.7656
Hemiarthroplasty		3 (9.4%)	4 (20%)	0.4076
Reverse Total Shoulder Arthroplasty		12 (37.5%)	10 (50%)	0.4027
Surgeon Fellowship Training				
Trauma		8 (25)	2 (10)	0.5069
Shoulder		21 (65.63)	16 (80)	
Trauma/Shoulder		1 (3.13)	0	
Other		2 (6.25)	2 (10)	
Operative Duration (minutes)		187 (IQR: 131-228.5) Min-max = 10–338	194.5 (IQR: 157-220.5) Min-max = 54–298	0.6181
Average Estimated Blood Loss (cc)		200 (IQR: 175–350) Min-max = 0-1100	250 (IQR: 150–350) Min-max = 50–600	0.5552

**Table 3:** Post-surgical outcomes in patients with surgery ≤ 5 vs. > 5 days from time of presentation

Measure	Surgery > 5 days from presentation (*n* = 52)		*p*-value
	No(*n* = 32 Injuries)	Yes (*n* = 20 Injuries)	
ICU Admission Postop	1 (3.1%)	2 (10%)	0.5511
Length of Stay (days)	5.0 (IQR 4.0–9.0) Min-max = 0–32	5.5 (IQR 2.0–16.0) Min-max = 2–47	0.7985
Reoperation (n, %)	4 (12.5%)	1 (5.0%)	0.6374
Number of Reoperations 0 2	28 (87.5%) 1 (9.38%)	19 (95.0%) 0 (0.0%)	0.4859
2	1 (3.13%)	1 (5.0%)	
Time to Reoperation (days)	91 (IQR 8-369) Min-max = 5-1079	21 (*n* = 1)	–
Wound Complications	0	1 (4.8%)	0.3846
Residual Neurologic injury	3 (9.38%)	1 (5.00%)	1.0000
EMG Acquisition	2 (6.25%)	0	0.5173
Neurologic Intervention			
Expectant Management	6 (18.75)	3 (15.00)	1.0000
Gabapentin None	1 (3.13) 25 (78.13)	0 17 (85.00)	
Mortality	4 (12.5%)	1 (5.0%)	0.6374
Time to Mortality (days)	75.5 (56.5-298.5) Min-max = 47–512	430 (*n* = 1)	–
Follow-up (months)	5.9 (IQR 2.8–12.7) Min-max = 0.9–42.2	5.0 (IQR 2.1–15.5) Min-max = 0.6–45.7	0.5103

## Discussion

There remains a paucity of literature examining the impact of operative timing on rates of neurologic injury following proximal humerus fracture dislocations. At our institution, we attempt to address proximal humerus fracture dislocations in an expeditious manner within 3–5 days pending specialist availability, operating room access, and patient medical clearance. We further advocate for operative intervention in patients presenting with neurologic deficits as soon as medically optimized. For this exploratory study, 5 days represents a logical cutoff to evaluate the impact of operative timing on patient outcomes. This exploratory study was unable to detect a difference in rates of new onset neurologic injury in the preoperative waiting period for patients who underwent operative intervention before or after 5 days from injury, however is significantly limited by the rarity of the primary outcome with only 1 patient in the cohort experiencing a preoperative neurologic change. No difference was identified in rates of residual neurologic deficit, reoperation, and mortality between groups. Collectively, these results add to the limited literature examining the optimal timing for management of these injuries by shoulder and trauma specialists and the impact operative delays have on neurologic injury development. Due to the underpowered nature of this study, larger studies should be performed to better define the safety of operative delays. The authors still advocate for early intervention for patients with a neurologic deficit or progressive deficit when the patient is cleared and the appropriate specialist and operating room is available.

Overall rate of neurologic injury of 14.0% falls within the previously reported range of 6.2 to 67% [11–13]. Through EMG and intraoperative neuromonitoring of proximal humerus fractures, it has been noted that neurologic injury frequently goes unrecognized on clinical exam, with 16% of nerve injuries being undiagnosed [11, 12]. The lack of reliability on clinical exam combined with 5.3% of our cohort having an unknown exam, suggests that the true rate of neurologic injury may be underestimated. While there is limited literature examining changes in neurologic exam preoperatively in relation to surgical timing, our series indicates an overall low incidence of new neurologic injury in the preoperative period regardless of time to operation with a rate of 0% and 5.0% for patients undergoing operation ≤ and > 5 days, respectively. Residual neurologic deficit for both cohorts was also similar between groups at 9.4% and 5.0%. The rate of residual neurologic deficit was noted to be higher than prior literature, suggesting that the combination of proximal humerus fracture and dislocation is a more traumatic mechanism to the brachial plexus than proximal humerus fractures alone [11]. While timing to operative intervention failed to detect a difference in new neurologic injury, diagnosis and management of neurologic injury in proximal humerus fractures are essential to optimize due to the functional limitations imposed by neurologic injuries that can contribute to capsular stiffness, persistent weakness of shoulder muscles, neuropathic pain, and poor functional outcomes [11]. The authors propose timely surgical intervention for medically optimized patients who have a proximal humerus fracture dislocation with an associated neurologic deficit requiring surgical intervention.

This study failed to detect a difference in rates of reoperation or mortality for patients undergoing operative intervention ≤ or > 5 days after sustaining a proximal humerus fracture with glenohumeral dislocation, however is also limited by the lack of statistical power. In a retrospective review of 30 proximal humerus fracture dislocations by Schnetzke et al., surgery within 48 h of injury was found to have a decreased risk of AVN and revision surgery [24]. While overall rate of AVN was low and similar between groups, assessment of AVN was limited in our cohort secondary to the high rate of index arthroplasty and short follow-up. In the absence of dislocation, Demir et al. highlights that hemiarthroplasty performed within 6–10 days after fracture was associated with a better Western Ontario Osteoarthritis of the Shoulder Index (WOOS), while patients with a delay of greater than 10 days demonstrated a progressive inverse relationship with WOOS outcomes in addition to lower patient satisfaction [14]. In the comparison of RTSA for proximal humerus fractures at 0–6 weeks versus 7–52 weeks by Parel et al., patients undergoing RTSA at 7–52 weeks were noted to have increased odds of revision within 2 years specifically for dislocation, periprosthetic fracture, and PJI [15]. Schirren et al. reported on a case series of anterior proximal humerus dislocations in which ORIF was performed at mean of 0.1 +/-1.3 days, hemiarthroplasty at 2.8 +/- 6.7 days, and RTSA at 4.1 +/-2.8 days following admission [8]. These times to intervention for proximal humerus fracture dislocations are notably shorter than those reported in other studies highlighting the need to further examine the optimal timing for proximal humerus fractures associated with glenohumeral dislocation.

## Limitations

This study has several limitations. The lack of long-term follow-up limits assessment and is inadequate for conclusions regarding secondary outcomes such as humeral head AVN, long term neurologic recovery and arthroplasty survival, both of which may be intimately associated with time from presentation to operative intervention. Similar to the optimal timing of reduction for the native hip and eventual AVN, future larger studies should work to define this time point for reduction of proximal humerus fractures with glenohumeral dislocations [25–28]. This study may be underpowered to make conclusions about the impact of operative timing on development of neurologic injury and postoperative outcomes specifically rates of AVN and reoperation and is at substantial risk of Type II error. While the authors recognize the severity of AVN, the relationship to neurologic injury may be more critical, as this may preclude future arthroplasty contributing to substantial loss of function, while precluding a salvage option for patients who experience AVN. Neurologic injury at presentation may also have led to earlier intervention confounding the outcomes of this study. However, injury severity of patients and the need for medical clearance frequently delayed operative interventions. With the retrospective nature and lack of prior studies examining changes in neurologic exam, no power analysis was completed. Given low sample size and inability to draw meaningful conclusions in heterogenous treatment groups, patient reported outcomes were not analyzed. The primary outcome of new onset neurologic injury in the preoperative waiting period was rare in both time cohorts, with only 1 event among 52 patients (1.9%), occurring in the delay group (0/32 (0%) vs. 1/20 (5.0%), *p* = .385). Consequently, this comparison was underpowered (~ 12% power). Given the available sample sizes, only a difference of ~ 30% would have had 80% power to be detectable. Therefore, the lack of statistical significance should be interpreted with caution. The threshold of 5 days for this study may further limit its generalizability, as there is limited literature specifically analyzing the impact of time to operation on neurologic injury in the preoperative waiting period. The authors used 5 days as a cutoff as this seemed to be a worst case scenario at our institution for access to specialized care or operating room time for surgeries such as shoulder arthroplasty. The rarity of the primary outcome of preoperative neurologic change further limits sensitivity analysis to be performed for the 5-day cutoff and represents an opportunity for larger studies. This study was unable to control for confounding factors such as selection bias and indications for timing of surgical intervention influenced by preoperative factors that may limit the conclusions regarding the impact of operative timing. The authors advocate for operative intervention as soon as a patient presenting with a neurologic deficit is medically optimized for surgery, which may have led to the increased proportion of patients with a presenting neurologic deficit being in the ≤ 5 days cohort. There is also the risk that the proportion of patients with unknown neurologic exam at time of presentation may have led to under recognition of neurologic injuries in the delayed group. Finally, there were the inherent limitations to any retrospective review utilizing the EMR. Objective information regarding neurologic injury was limited to physical exams documentation, as EMGs are not routinely obtained preoperatively which may be confounding and underestimated rates of neurologic injury. However, as EMGs are not routinely obtained preoperatively, neurologic exam remains the standard for guiding decisions in this patient population.

## Conclusions

New onset neurologic injury in the preoperative waiting period for operative intervention of proximal humerus fracture dislocations was a rare occurrence within this exploratory retrospective study with only 1 patient experiencing a change in neurologic exam in the > 5 days to intervention cohort. While no difference was detected in rates of new onset neurologic injury in the preoperative waiting period between patients undergoing operative intervention ≤ or > 5 days following a proximal humerus fracture with glenohumeral dislocation, the rarity of preoperative neurologic change in this exploratory study limits definitive statistical conclusions regarding the safety of waiting for operative intervention for these injuries. Due to the underpowered nature of this study, future larger studies are needed to corroborate these findings and better examine avascular necrosis, reoperation, and patient outcomes.

## Data Availability

No datasets were generated or analysed during the current study.
